# Inhibition of insulin resistance by PGE1 via autophagy-dependent FGF21 pathway in diabetic nephropathy

**DOI:** 10.1038/s41598-017-18427-2

**Published:** 2018-01-08

**Authors:** Wei Wei, Xing-Rong An, Shi-Jie Jin, Xiao-Xue Li, Ming Xu

**Affiliations:** 1 Department of Clinical Pharmacy, School of Basic Medical Sciences and Clinical Pharmacy, China Pharmaceutical University, Nanjing, 210009 China; 2 School of Pharmacy, Zhejiang Chinese Medical University, Hangzhou, 311400 China; 3 Department of Pathology, Medical School of Southeast University, Nanjing, 210009 China

## Abstract

Insulin resistance is a critical process in the initiation and progression of diabetic nephropathy (DN). Alprostadil (Prostaglandin E1, PGE1) had protective effects on renal function. However, it is unknown whether PGE1 inhibited insulin resistance in renal tubule epithelial cells via autophagy, which plays a protective role in DN against insulin resistance. Insulin resistance was induced by palmitic acid (PA) in human HK-2 cells, shown as the decrease of insulin-stimulated AKT phosphorylation, glucose transporter-4 (GLUT4), glucose uptake and enhanced phosphorylation of insulin receptor substrate 1(IRS-1) at site serine 307 (pIRS-1ser307) and downregulated expression of IRS-1. Along with less abundance of p62, autophagy markers LC3B and Beclin-1 significantly increased in HK-2 cells exposed to PA. Such abnormal changes were significantly reversed by PGE1, which mimicked the role of autophagy gene 7 small interfering RNA (ATG7 siRNA). Furthermore, PGE1 promoted the protein expression of autophagy-related fibroblast growth factor-21 (FGF21), which alleviated insulin resistance. Results from western blotting and immunohistochemistry indicated that PGE1 remarkably restored autophagy, insulin resistance and the FGF21 expression in rat kidney of type 2 diabetes mellitus (T2DM). Collectively, we demonstrated the potential protection of PGE1 on insulin resistance in renal tubules via autophagy-dependent FGF21 pathway in preventing the progression of DN.

## Introduction

Diabetic nephropathy (DN) is one of the major microvascular complications of diabetes to lead to end-stage renal disease^1^. Insulin resistance is of importance in the initiation and progression of DN^2^. It was reported that the patients with type 2 diabetes mellitus (T2DM) who develop DN have been shown to be more insulin resistant than those who do not. Insulin resistance is closely associated with microalbuminuria which is one of the main symptoms of DN^3–5^. Given that tubular functional and morphological changes precede the onset of microalbuminuria in early DN, it is worth studying whether tubule epithelial cells are novel insulin-sensitive cells.

Autophagy is a major catabolic pathway by which mammalian cells degrade macromolecules and organelles to maintain intracellular homeostasis^6^. Autophagy is involved in the pathogenesis of DN as a novel therapeutic target. In the field of kidney research, proximal tubular cells are a focus of autophagy studies. Under basal conditions, autophagy activity maintains in very low level in proximal tubular cells. The presence or activation of autophagy plays a protective role in DN against insulin resistance, however, excessive autophagy results in various renal tubular injury^7^.

Therefore, there are great potential clinical implications in understanding the mechanisms underlying insulin resistance in renal dysfunction by pharmacological interventions. Alprostadil (Prostaglandin E1, PGE1) is one of hormones synthesized by most tissues^8^ to regulate blood flow in variety of organs^9,10^. In the clinical, PGE1 may have positive effects on DN by improving renal blood circulation, decreasing albuminuria and lessening proteinuria^11,12^. It has been not reported that PGE1 has the potential protection on insulin resistance in renal tubule epithelial cells via autophagy.

The present study hypothesized that the inhibition of insulin resistance is an important mechanism leading to the preventive effects of PGE1 on DN. Cellular insulin resistance in human proximal tubule epithelial cell line (HK-2 cells) was induced by palmitic acid (PA), a most common saturated free fatty acids (FFA), which are thought to be the major cause of insulin resistance in T2DM^13,14^. T2DM is clearly an insulin-resistant state which is produced in rats fed with high fat diet (HFD) combined with low dose streptozotocin (STZ) administration. Our results indicated that PGE1 inhibited insulin resistance and thereby improved renal dysfunction in T2DM rats. We also demonstrated that this decrease of autophagy and consequent upregulation of downstream molecular fibroblast growth factor-21 (FGF21) resulted in the recovery of insulin resistance by PGE1. These studies thus revealed a potential mechanism for the beneficial action of PGE1 in diabetic renal complication.

## Results

### PGE1 ameliorated insulin resistance induced by PA in HK-2 cells

**Figure 1:**
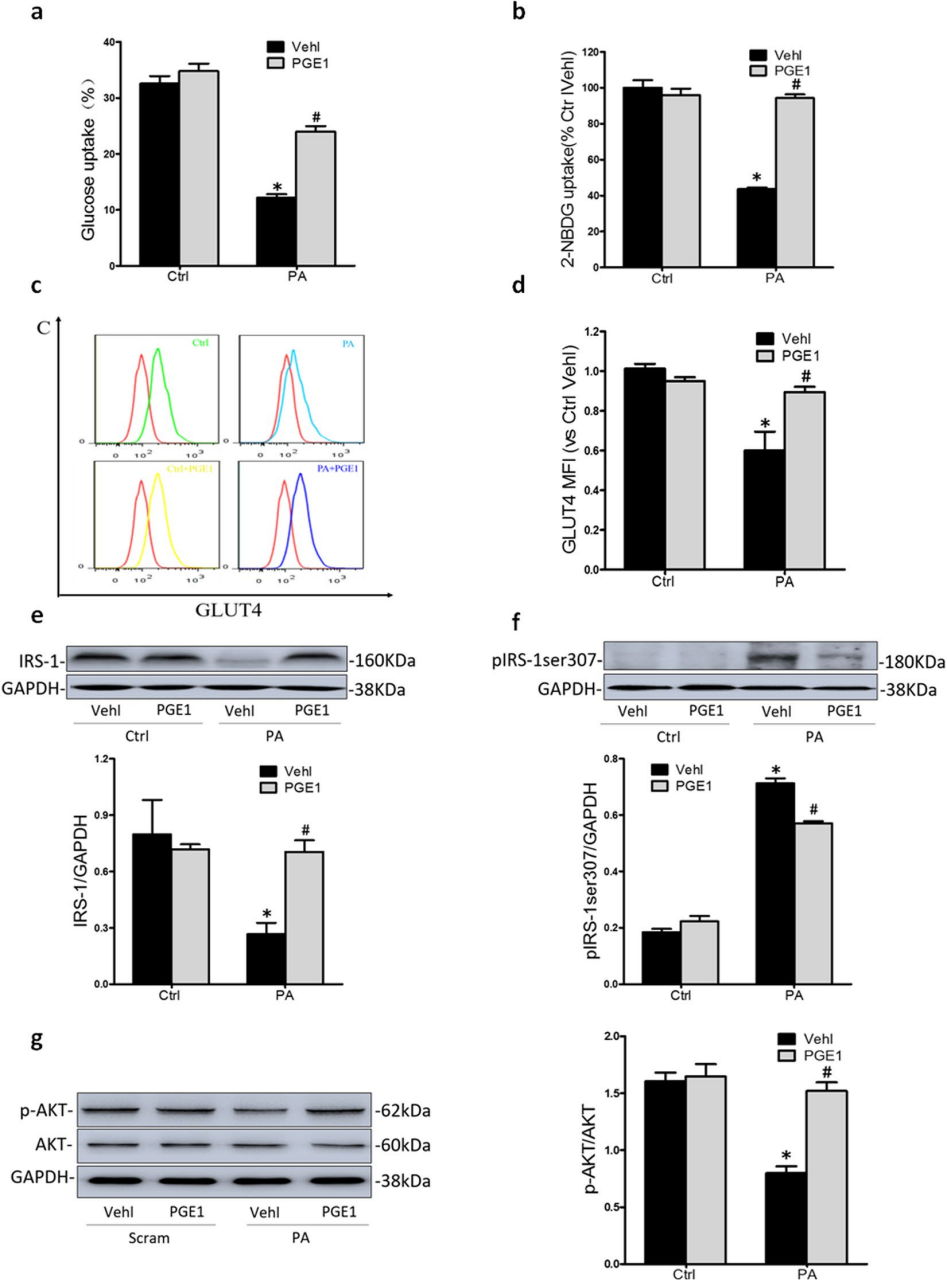
PGE1 ameliorated PA-induced insulin resistance in HK-2 cells. HK-2 cells were incubated with PA (0.5 mM) for 48 h, which was treated with PGE1 (0.1 ng/ml) and insulin (100 nM) for 24 hours. HK-2 cells in normal medium without PA were regarded as control group. (**a**) Summarized data showing the ratio of glucose uptake in HK-2 cell medium supernatant. (**b**) Summarized data showing 2-NBDG uptake in HK-2 cell. (**c**,**d**) Frequency histogram of GLUT4 in the membranes and summarized mean fluorescence intensities (MFI) showing the expression of GLUT4 in plasma membrane by flowcytometry. Representative Western blot gel documents and summarized data showing the protein expression of IRS-1 (**e**) and pIRS-1ser307 (**f**) in HK-2 cells. *P < 0.05 vs. Ctrl; ^**#**^ *P* < 0.05 vs. PA treated group (n = 3).

We first confirmed that PA could induce insulin resistance in HK-2 cells. MTT assay showed that there was no significant difference in cell viability after treatment with PA (0.5 mM) or PGE1 (0.1 ng/ml) for 48 h. As shown in Fig. 1a,b, insulin-stimulated glucose uptake was measured by glucose oxidase-peroxidase (GOD-POD) assay and 2-NBDG uptake which detected the extracellular glucose surplus and intracellular glucose intake, respectively. PA decreased glucose uptake in HK-2 cells as measured. At the same time, PA significantly decreased the insulin-stimulated glucose transporter-4 (GLUT4) translocation, the protein expression of insulin receptor substrate-1(IRS-1) and insulin signaling molecule AKT phosphorylation, but induced the phosphorylation of insulin receptor substrate-1 at site serine 307 (pIRS-1ser307) to verify insulin resistance in HK-2 cells (Fig. 1c–g).

We further found that the indexes above of insulin resistance were significantly restored by the pretreatment with PGE1. These data suggested that PGE1 ameliorated PA-induced insulin resistance in HK-2 cells through regulating the activity or protein expression of GLUT4, IRS-1and AKT.

### PGE1 inhibited autophagy activation induced by PA

**Figure 2:**
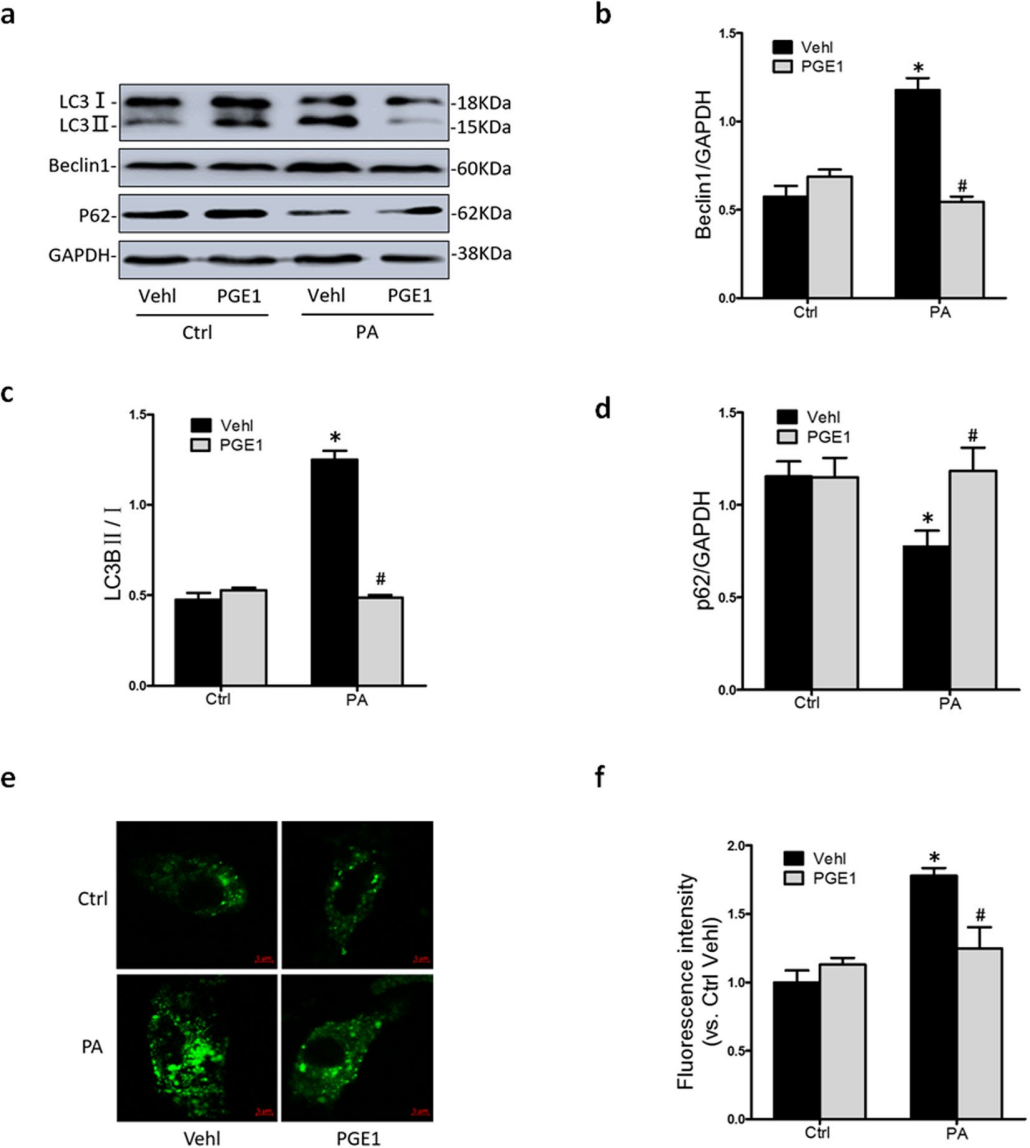
PGE1 inhibited abnormal elevation of autophagy induced by PA. HK-2 cells were incubated with PA (0.5 mM) for 48 h, which was treated with PGE1 (0.1 ng/ml) for 24 hours. HK-2 cells in normal medium without PA were regarded as control group. Representative Western blot gel documents (**a**) and summarized data (**b**,**c**,**d**) showing the protein expression of LC3B, Beclin-1 and p62 in HK-2 cells. Typical fluorescent images (**e**) show the MDC staining HK-2 cells by confocal microscope and summarized data (**f**) showed the fluorescence intensity. *P < 0.05 vs. Ctrl; ^#^ *P* < 0.05 vs. PA treated group (n = 3).

It was reported that PA can induce autophagy in the different tissues to cause apoptosis^15–17^. To test the effects of PA on autophagy in HK-2 cells, autophagy-related markers were examined by western blotting. Microtubule-associated protein 1 light chain 3 beta (LC3B) is an autophagy marker protein, which is recruited to autophagosomal membranes during the formation of autophagosomes. It was found that the LC3B II level significantly increased in HK-2 cells exposed to PA. Similarly, PA led to an increase of Beclin-1 protein, which is required for the initiation of the autophagasome formation. The abundance of p62, a selective substrate of the autophagy degrading pathway was less in HK-2 cells pretreated with PA, suggesting that more p62 protein was degraded due to enhanced autophage by PA. Such changes above were significantly reversed by PGE1 treatment (Fig. 2a–d). These results suggested that PGE1 inhibited PA-induced autophagy formation and activation in HK-2 cells.

In order to further demonstrate PGE1 regulates autophage, MDC, a fluorescent compound was used for the selectively labeling of autophagic vacuoles. Green punctuates or patches in cytoplasmic and perinuclear regions by confocal microscope represented autophagic vacuoles. Under control conditions, only a few green dots were detected, which was markedly increased due to PA treatment to show more autophagosomes were formed or accumulated in HK-2 cells (Fig. 2e,f). PGE1 inhibited the accumulation of autophagic vacuoles induced by PA, which confirmed the inhibitory role of PGE1on autophagy formation and activation.

### The inhibition of autophagy alleviated insulin resistance

**Figure 3:**
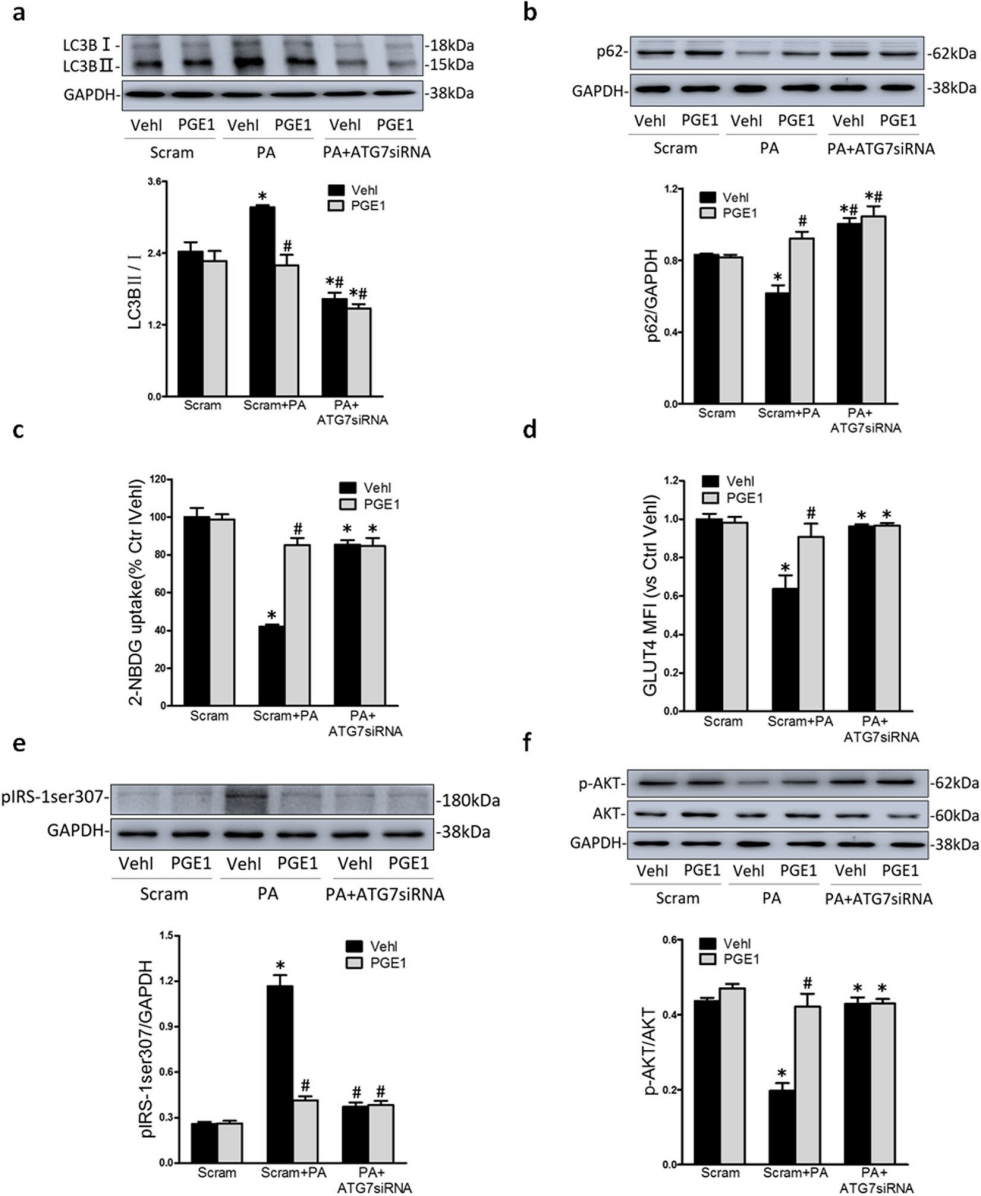
The inhibition of autophagy alleviated insulin resistance. HK-2 cells were incubated with PA (0.5 mM) for 48 h, which was treated with PGE1 (0.1 ng/ml) and insulin (100 nM) in the presence or absence of the transfection of ATG7 siRNA. HK-2 cells in normal medium without PA were regarded as control group. Representative Western blot gel documents and summarized data showing the protein expression of LC3B (**a**) and p62 (**b**). Summarized data showing 2-NBDG uptake (**c**) and MFI for expression of GLUT4 in plasma membrane by flowcytometry (**d**). Representative Western blot gel documents and summarized data showing the protein expression of pIRS-1ser307 (**e**) and p-AKT (**f**) in HK-2 cells. *P < 0.05 vs. Ctrl; ^#^ *P* < 0.05 vs. PA treated group (n = 3).

To investigate the relationship between autophagy and PA-induced insulin resistance, HK-2 cells were treated with autophagy key gene 7 small interfering RNA (ATG7 siRNA) and a specific autophagy inhibitor, 3-methyladenine (3-MA, 5 mM)^18^. As showed in Fig. 3a,b, the expression of LC3B II was inhibited and the accumulation of p62 was increased by ATG7 siRNA and 3-MA (not shown). Thus, ATG7 siRNA and 3-MA was regarded as an autophagy inhibitor in current study.

Importantly, ATG7 siRNA significantly increased insulin-stimulated glucose uptake in HK-2 cells exposed in PA for 48 hours (Fig. 3c). The insulin-stimulated phosphorylation of IRS-1 at site Ser-307 and AKT and GLUT4 translocation were reversed by the transfection with ATG7 siRNA which mimicked the role of PGE1 (Fig. 3d–f). Furthermore, PGE1 combined with ATG7 siRNA showed no additive effects on these indexes above of insulin resistance. Therefore, these results indicated that the inhibition of PA-induced activation of autophagy could contribute to insulin resistance in HK-2 cells.

### Inhibition of autophagy induced the expression of FGF21 in PA-treated HK-2 cells

**Figure 4:**
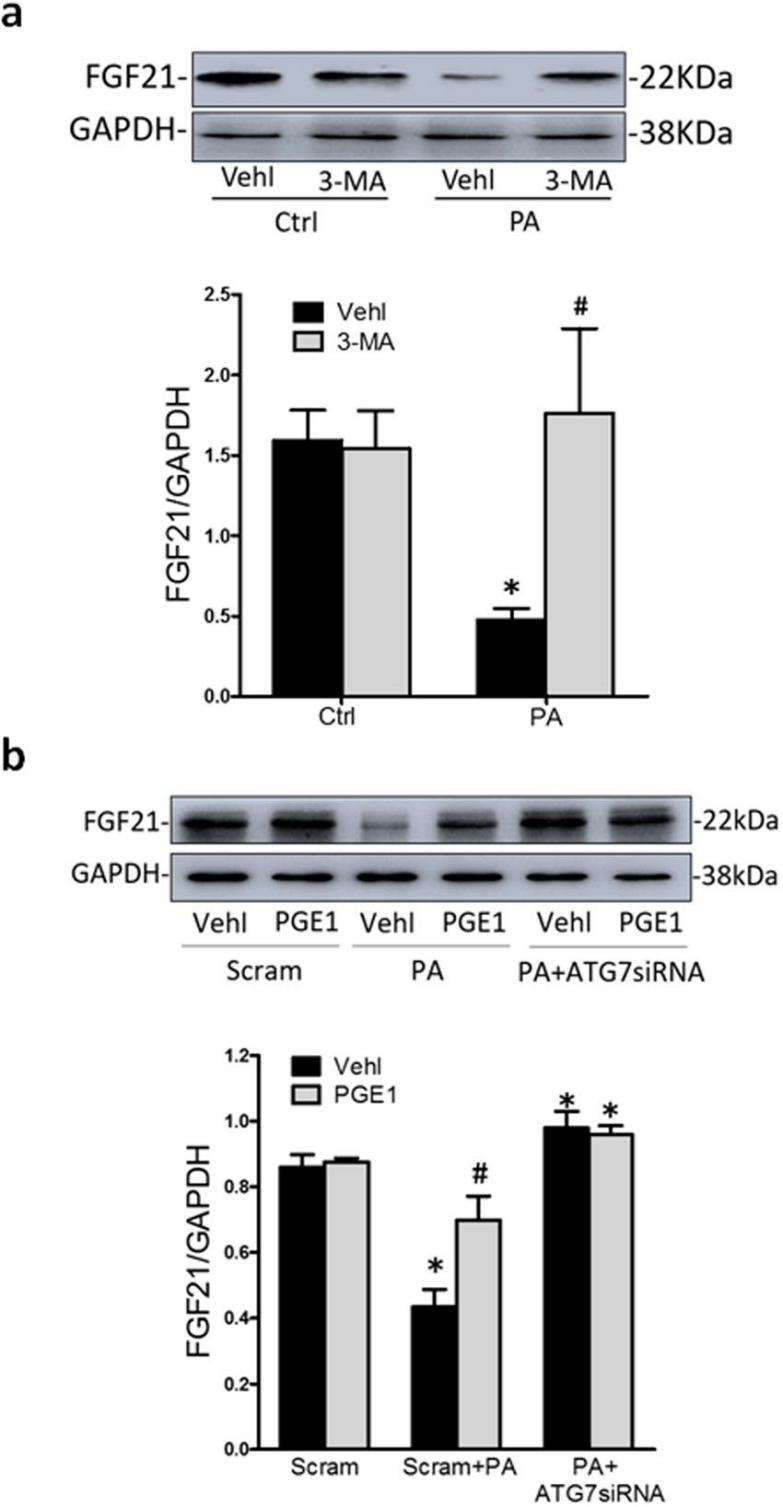
Inhibition of autophagy induced the expression of FGF21 in PA-treated HK-2 cells. HK-2 cells were incubated with PA (0.5 mM) for 48 h, which was treated with PGE1 (0.1 ng/ml) in the presence or absence of the transfection of ATG7 siRNA or pretreatment of 3-MA (5 mM). HK-2 cells in normal medium without PA were regarded as control group. Representative Western blot gel documents and summarized data showing the protein expression of FGF21 (**a**,**b**) in HK-2 cells. *P < 0.05 vs. Ctrl; ^#^ *P* < 0.05 vs. PA treated group (n = 3).

It was reported that the expression of FGF21 in skeletal muscle is promoted by autophagy deficiency^19^. Here, we first found that FGF21 was expressed in HK-2 cells and then examined the role of autophagy on FGF21 by ATG7 siRNA and 3-MA. The results showed that PA decreased FGF21 expression in HK-2 cells, which was significantly reversed by inhibiting PA-induced autophagy (Fig. 4a,b). Similarly, PGE1 also promoted the expression of FGF21 protein, and PGE1 combined with the ATG7 siRNA showed no additive effects on the expression of FGF21 in PA-treated HK-2 cells (Fig. 4b). These data provided key evidence that inhibiting autophagy was responsible for the enhancement of FGF21 by PGE1 in PA-treated HK-2 cells.

### FGF21 ameliorated insulin resistance in HK-2 cells

**Figure 5:**
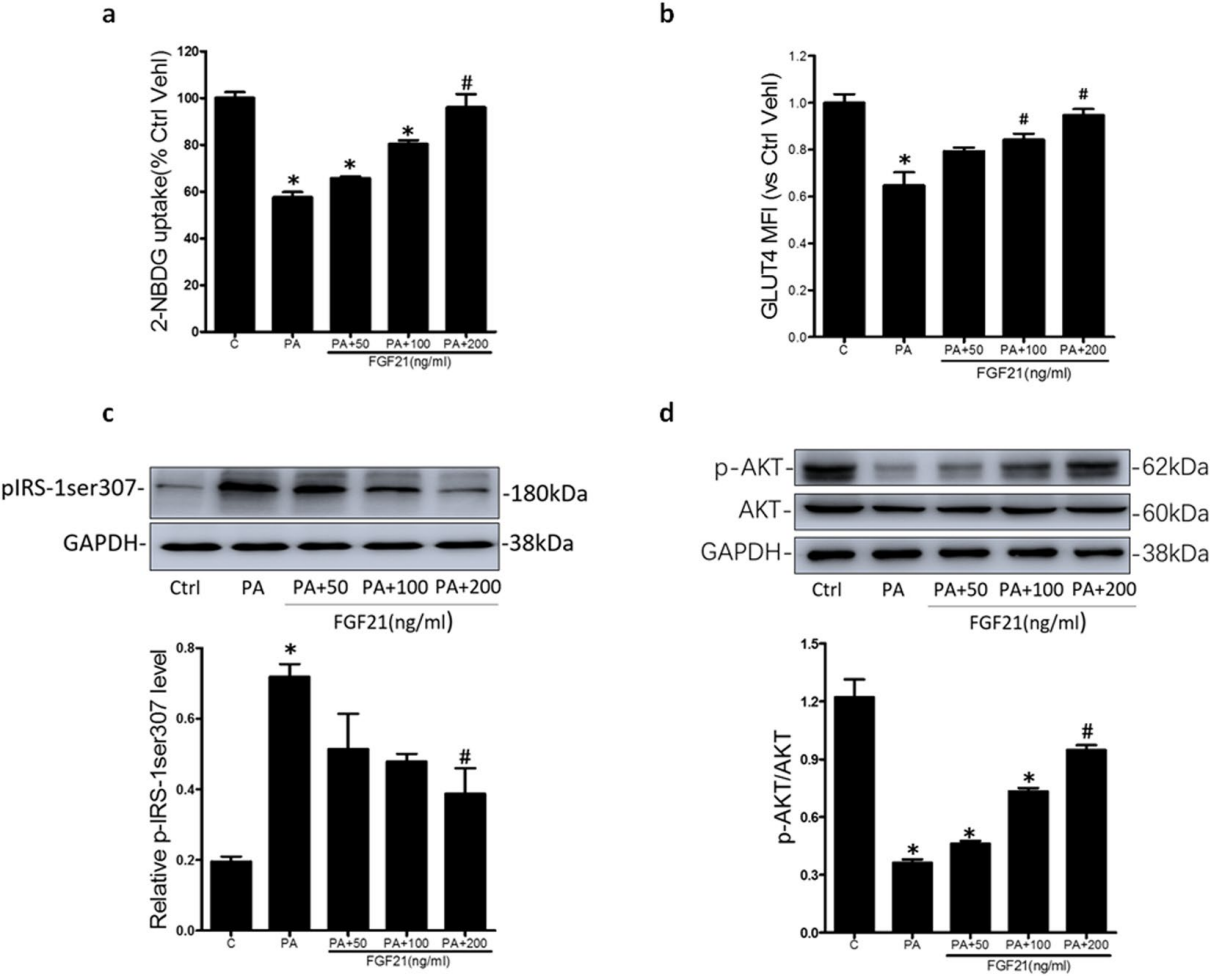
FGF21 ameliorated insulin resistance in HK-2 cells. HK-2 cells were pretreated with PA (0.5 mM) for 48 h, and stimulated by 100 nM insulin in the presence of FGF21 (0, 50 ng/ml, 100 ng/ml, 200 ng/ml). Summarized data showing 2-NBDG uptake (**a**) and MFI for expression of GLUT4 in plasma membrane by flowcytometry (**b**). Representative Western blot gel documents and summarized data showing the protein expression of pIRS-1ser307 (**c**) and p-AKT (**d**) in HK-2 cells. *P < 0.05 vs. Ctrl; ^#^ *P* < 0.05 vs. PA treated group (n = 3).

We further explored the effect of FGF21 on insulin resistance. HK-2 cells were treated with gradient concentration of FGF21 (0, 50 ng/ml, 100 ng/ml, and 200 ng/ml). As shown in Fig. 5, compared with PA alone treated group, glucose uptake, GLUT4 translocation and p-AKT expression dose-dependently increased due to the pretreatment with FGF21. Conversely, the expression of pIRS-1ser307 dose-dependently decreased in the presence of FGF21 (Fig. 5c). These data suggested that FGF21 indeed ameliorated insulin resistance in HK-2 cells.

### PGE1 alleviated insulin resistance through FGF21 in HK-2 cells

**Figure 6:**
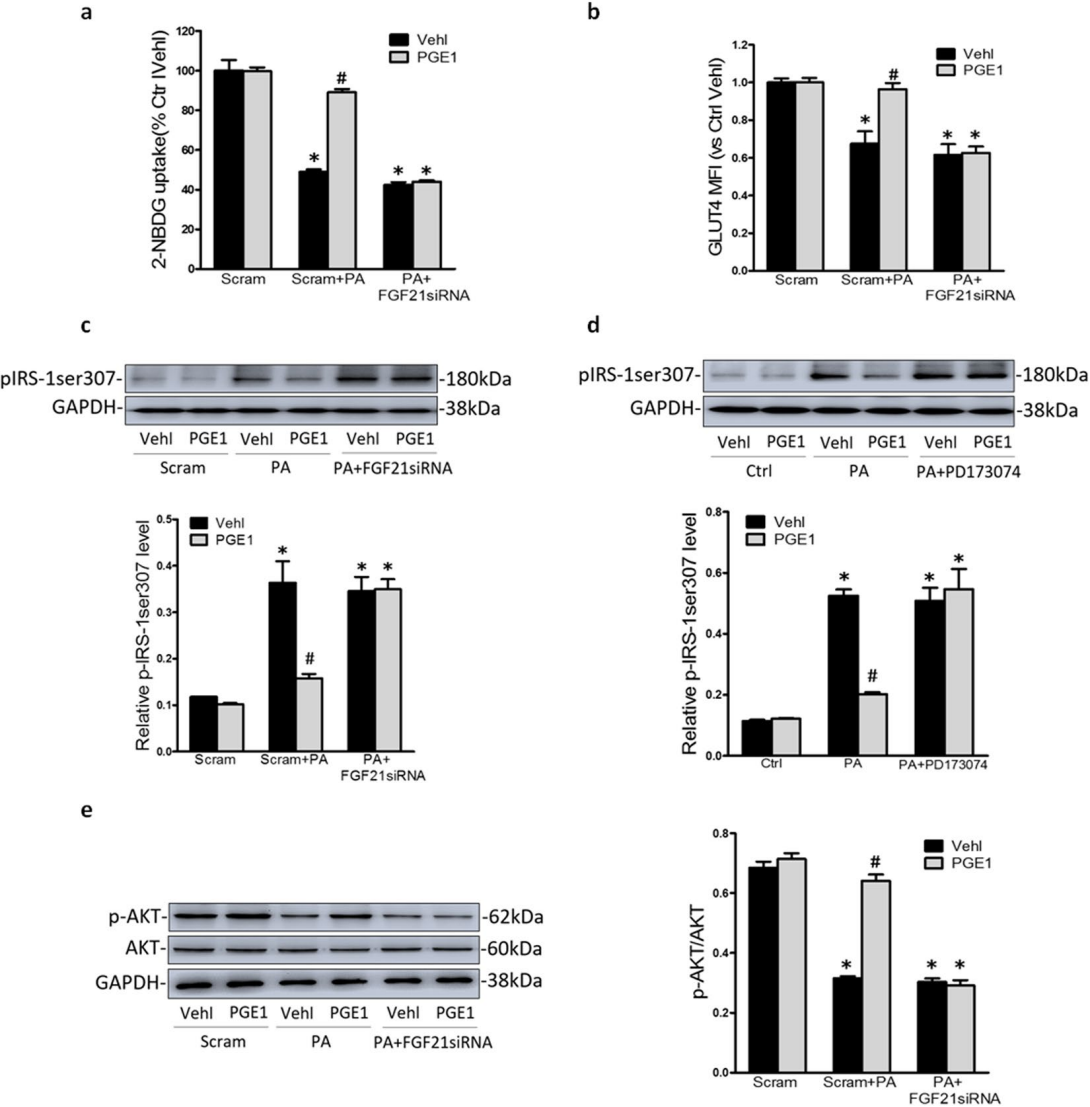
PGE1 alleviated HK-2 cell insulin resistance through FGF21 signaling pathway. HK-2 cells were incubated with PA (0.5 mM) for 48 h, which was treated with PGE1 (0.1 ng/ml) and insulin (100 nmol/L) in the presence or absence of the transfection of ATG7 siRNA or PD173074. Summarized data showing -NBDG uptake (**a**) and MFI for expression of GLUT4 in plasma membrane by flowcytometry (**b**). Representative Western blot gel documents and summarized data showing the protein expression of pIRS-1ser307 (**c**,**d**) and p-AKT (**e**) in HK-2 cells. *P < 0.05 vs. Ctrl; ^**#**^ *P* < 0.05 vs. PA treated group (n = 3).

To investigate whether the amelioration of insulin resistance by PGE1 was related to FGF21, FGF21 siRNA and FGFR inhibitor PD173074 were respectively used to block FGF21 signaling pathway. As shown in Fig. 6, the expression of insulin-stimulated glucose uptake, GLUT4 translocation and the expression of pIRS-1ser307 and p-AKT showed no significant difference between PA alone treated HK-2 cells and PA treated HK-2 cells transfected with FGF21 siRNA or treated with PD173074. However, compared with HK-2 cells incubated with PA plus PGE1, the indexes above were markedly reversed due to the transfection with FGF21 siRNA or the treatment with PD173074. The results demonstrated that blocking FGF21 signal is indeed responsible for the amelioration of insulin resistance by PGE1 in HK-2 cells.

### PGE1 improved renal function and pathology in T2DM rats

**Figure 7:**
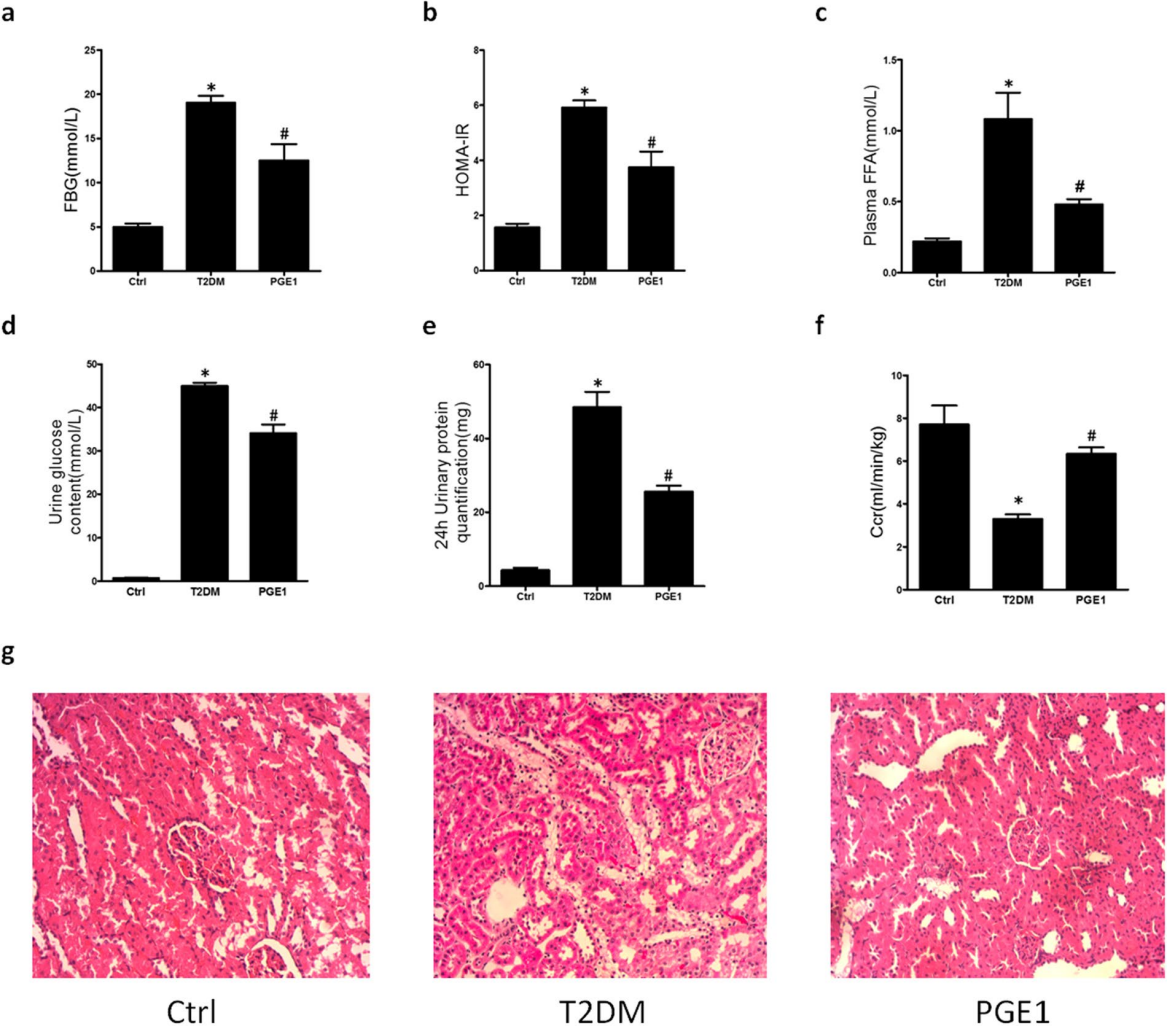
PGE1 improved insulin resistance, renal function and pathology in diabetic rats. Male Sprague-Dawley rats were fed HFD and a low dose of STZ (40 mg/kg) to develop a rat model of type2 diabetes. Diabetic rats were intervened with PGE1 (20 μg/kg/d) for 4 weeks. Summarized data showing the FBG (**a**), HOMA-IR (**b**) and FFA (**c**), urine glucose content (**d**), urine protein quantification (**e**) and Ccr (**f**). (**g**) Representative images showing HE staining morphological change. *P < 0.05 vs. Ctrl; ^**#**^ *P* < 0.05 vs. diabetic rat group (n = 6).

Following an injection of STZ in rats with HFD, FBG and FFA was significantly elevated relative to control group (Fig. 7a,c). Homeostasis model assessment of insulin resistance (HOMA-IR) in T2DM rats was significantly increased, compared to control rats (Fig. 7b). These changes were along with an increase in urine glucose content and microproteinuria in 24 h and a decrease in endogenous creatinine clearance (Ccr) in rats treated with HFD/low STZ (Fig. 7d–f). Following PGE1 (20 μg/kg/d) administration, FBG, HOMA-IR, FFA, urine glucose, and microproteinuria in 24 h were significant suppressed as well as an increase of Ccr.

The renal pathology of rats was studied by HE staining (Fig. 7g). The normal appearance of renal histological structure was seen in control rats. In rats fed HFD/low STZ, the swellings of the renal tubules were significantly exacerbated contributing to the pathological basis for the renal failure. After four weeks PGE1 injection, the vacuolar degeneration of proximal tubule epithelial cells was significantly ameliorated. The results suggested that PGE1 is promising to improve the function of renal tubules in DN.

### PGE1 ameliorated renal insulin resistance in T2DM rats

**Figure 8:**
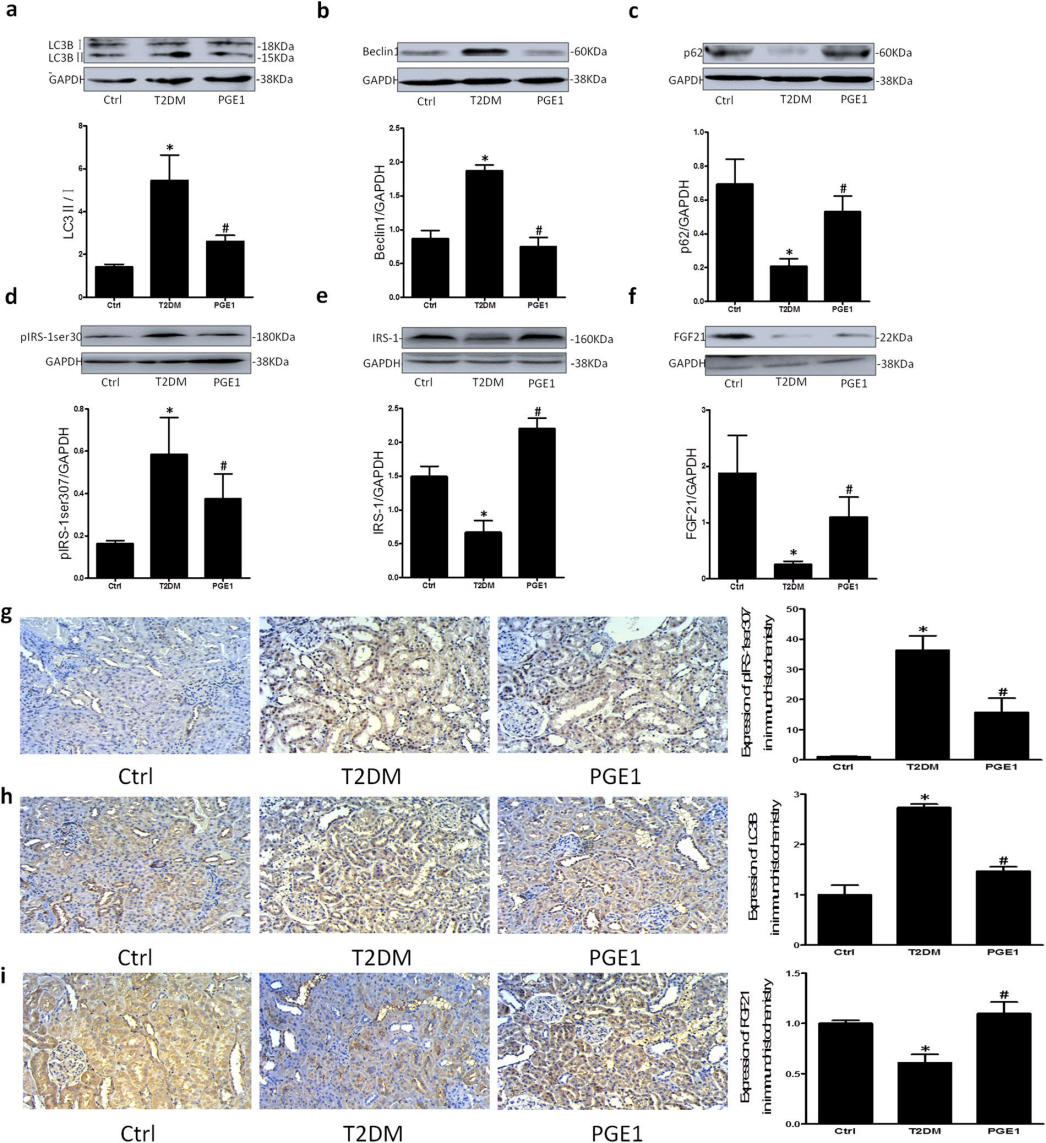
PGE1 ameliorated renal insulin resistance via autophagy-dependent FGF21 in diabetic rats. Representative Western blot gel documents and summarized data showing the protein expression of LC3B (**a**), Beclin-1 (**b**), p62 (**c**), pIRS-1ser307 (**d**), IRS-1 (**e**) and FGF21 (**f**) in diabetic rat kidney. Representative immunohistochemistry images and summarized data of pIRS-1ser307 (**g**), LC3B (**h**) and FGF21 (**i**) in rat kidney. *P < 0.05 vs. Ctrl; ^**#**^ *P* < 0.05 vs. diabetic rat group (n = 3).

To confirm the role of PGE1 on DN and mechanisms, we examined the makers of autophagy (Fig. 8a–c), insulin resistance (Fig. 8d,e), and the FGF21 expression (Fig. 8f) in rat kidney by western blotting. A remarkable increase in LC3B and beclin1 and decrease of p62 expression in rat kidney were elicited followed by STZ and HFD. After intervened with PGE1, the activation in autophagy was significantly reversed. At the same time, PGE1 restored the abnormal phosphorylation of IRS-1 at the site of serine 307 and IRS-1 expression in T2DM rat kidney. Moreover, the protein expression of FGF21 in diabetic kidney increased following interventions with PGE1.

Similar results were found in renal immunohistochemistry (Fig. 8g–i). The positive deposits of LC3B and pIRS-1ser307 were mainly located along with the renal tubules. In control group, no significantly visible precipitation of LC3B and pIRS-1ser307 was found in the renal tubular structure but a definite amount of LC3B and pIRS-1ser307 protein could be noted in T2DM rats. The intensified precipitation of LC3B and pIRS-1ser307 proteins was significantly diminished following interventions with PGE1. Reversely, PGE1 markedly increased the intensified precipitation of FGF21 proteins in diabetic kidney. These results above *in vivo* demonstrated that PGE1 may ameliorate insulin resistance in renal tubule through autophagy-dependent FGF21 pathway, resulting in relieving the renal pathology of diabetic rats.

## Discussion

The present study for the first time demonstrated that renal tubule epithelial cells were novel insulin-sensitive cells and renal tubular insulin resistance is critical in the pathogenesis of DN. PGE1 increased FGF21 protein expression in HK-2 cells and rat kidney via inhibiting autophagy. Notably, the inhibition of insulin resistance by PGE1 resulted from the inactivation on autophagy, which was associated with recovery of T2DM-induced renal dysfunction.

Clinically, reducing proteinuria is considered a principal therapeutic target to improve renal outcomes in patients with DN. Angiotensin-converting-enzyme inhibitor and angiotensin-II receptor blocker may slow the progression of DN^20–22^. However, the onset of these drugs needs at least 4–5 years^23^ and the benefit seems limited^24^. Recent studies showed that PGE1 had protective effects on renal function by reducing vascular resistance in ischemia-reperfusion^25^, inhibiting the production of transforming growth factor-β (TGF-β) and interleukin-1 (IL-1) in immune-mediated glomerular disease^26^, and increasing microvessel density in acute toxic renal injury^27^. But it is currently unclear if PGE1 could be used for preventing the progression of DN. The main goal of the present study is to investigate whether PGE1 alleviates insulin resistance in proximal tubule epithelial cell against renal lesions and its underlying mechanisms. Palmitic acid (PA), a most common saturated FFA, restrains the insulin stimulated glucose transport to induce peripheral insulin resistance^28^. Here, we found that PGE1 promoted the uptake of glucose in HK-2 cells treated with PA, which is a possible mechanism to slow the progression of DN. Further, PGE1 recovered insulin -stimulated GLUT4 translocation and the phosphorylation of IRS-1 and AKT in HK-2 cells treated with PA. To our knowledge, these results for the first time provided evidences that HK-2 cells are novel insulin-sensitive cells and then PGE1 alleviates insulin resistance in HK-2 cells. Our results are similar with the report that PGE1 regulated metabolic function, promoted glucose uptake, maintain the homeostasis of glucose and glycogen in hepatocyte^29^.

Autophagy is considered to keep a cellular homeostatic status in various kinds of stress state, such as nutrient stress, hypoxia, ER stress, immune signals, redox stress, mitochondrial damage and so on^30^. Under normal circumstances, the basal autophagy level is very low in proximal tubule cells compared with that in podocytes^31^, but it is essential to keep cell homeostasis. Studies have shown that PA triggers autophagy^15,32^, which was demonstrated by our results that abnormally elevated autophagy was accompanied with insulin resistance in HK-2 cells treated with PA. In addition, PGE1 inhibited PA-induced autophagy to baseline levels in HK-2 cells which role was similar with ATG7 siRNA and autophagy inhibitor 3-MA. These results may imply that PGE1 may have a protective effect on diabetic proximal tubules injury through inhibiting excessive autophagy. Our results are similar with some previous studies that inhibition of autophagy increased cell viability in proximal tubule cells exposed in CoCl_2_, PA or high glucose^16,33,34^. Thus, the advantages of targeting autophagy for the treatment of DN is recommended in new drug research.

Recent studies have reported insulin resistance is connected with autophagy under various pathological conditions. It was reported that specific knockout of muscle ATG7 resulted in autophagy deficiency to prevent from HFD-induced insulin resistance^19^. Another study indicated that insulin resistance in peripheral insulin-sensitive tissues induced by ER stress was accompanied by anomalously elevated autophagy^35^. Consistently, our data demonstrated that the inhibition of autophagy by ATG7 siRNA and 3-MA ameliorated the insulin resistance in PA-treated HK-2 cells. Combined with the roles of PGE1 on autophagy and insulin resistance, we demonstrated that PGE1 had a protective effect on PA-induced insulin resistance through inhibiting excessive autophagy in HK-2 cells. Our results also revealed a new relation of between autophagy and insulin resistance in HK-2 cells, which is inconsistent with some previous reports. For example, autophagy-deficient mice showed a decrease in insulin sensitivity and a decrease in glucose utilization^36^. Knockout of ATG5 caused spontaneous glomerular lesions in mice podocytes in DN^37^. In addition, some studies reported that autophagy in proximal tubular cells plays a renoprotective role in various stages of proteinuric kidney diseases^38,39^. Thus, autophagy may play a double-edged sword role in insulin resistance and consequent renal pathological changes, which are dependent on DN stages, cell type and autophagy degree^40^.

Next, the present study identifies the mechanisms by which autophagy inhibited insulin resistance in HK-2 cells treated with PGE1. FGF21 is an endocrine protein to directly promote glucose uptake by increasing the expression of GLUT1 and IRS-1^41,42^. It was reported that the defective autophagy had a protective effect on insulin resistance in HFD-fed obese mice by promoting FGF21 expression^19^. We confirmed the results that inhibition of autophagy resulted in an increased expression of FGF21 protein in HK-2 cells, which mediated the inhibition of insulin resistance by PGE1. It was also reported that FGF21 markedly decreased urinary albumin excretion and ameliorated inflammation, fibrotic effect, oxidative stress as well as morphological lesions in DN^43,44^. Therefore, FGF21 has been considered as potential therapeutic agent for obesity and T2DM^45^.

We further confirmed the role of PGE1 and its mechanisms in T2DM rats produced by HFD/low STZ. The present study verified the protective effect of PGE1 on diabetic renal dysfunction. Accumulating evidences indicated that PGE1 reduces albuminuria mainly through improving haemodynamic changes including decreasing intraglomerular pressure and increasing kidney blood circulation^46,47^. Here, we surprisingly found that PGE1 inhibited insulin resistance and decreased the blood glucose in T2DM rats, which was closely associated with the improvement of renal dysfunction. In addition, the previous studies about PGE1 focused on the glomerular lesion in DN. Currently, HE staining found that PGE1 alleviated the pathological changes of renal tubular epithelium, suggesting that PGE1 may alleviate renal tubular lesion in DN. Our findings provide further evidences that PGE1 contributed to improve renal dysfunction in T2DM patients^48^. The renoprotective role of PGE1 has also been reported extensively in the treatment of ischemic renal reperfusion injury, toxic kidney injury, immune-mediated glomerular injury, and contrast-induced nephropathy, consequently resulting in slow progression of ESRD^27,49–51^. Thus, the present study provided new mechanisms for renoprotective role of PGE1 in clinical research.

Lastly, we examined autophagy and insulin resistance in rat kidney. Consistent with the results *in vitro*, the results from WB and immunohistochemistry suggested that the level of both autophagy and insulin resistance were formed and activated in the kidney of rats fed with by HFD/low STZ. PGE1 inhibited excessive autophagy and insulin resistance, and increased the expression of FGF21 in kidney tubule in T2DM rats. Combined with the results *in vitro*, PGE1 may ameliorate insulin resistance in proximal tubule through the suppression of autophagy and the upregulation of its downstream molecualar FGF21, consequently resulting in relieving the kidney dysfunction.

In conclusion, our data suggested that inhibition of autophagy and consequent improvement of insulin resistance are important determinants of renal dysfunction by PGE1 in DN, which provided new perception of the underlying molecular mechanisms of PGE1 protection on renal function in DN. Also, we identified autophagy and downstream FGF21pathway as potential therapeutic targets for DN.

## Materials and Methods

### Cells culture and reagents

Stable HK-2 cell lines are derived from human kidney tubule cells. HK-2 cells were cultured in DMEM/F12 (Gibco, lifetechnologies, China) with 10% fetal bovine serum (FBS, Gibco, lifetechnologies, USA), 100 U/ml penicillin and 100 mg/ml streptomycin, maintained at 37 °C with 5% CO_2_.

PA (Sigma) was conjugated with fatty acid-free bovine serum albumin (BSA) before added to cell culture medium according to the method described by Chavez *et al*.^52^. PA was dissolved in anhydrous ethanol completely at 200 mmol/L, then dissolved in DMEM/F12 containing 2% BSA, treated with ultrasonic at 55 °C until the solution was clear. The solution was filtered with 0.22 μm filter immediately. PGE1 was purchased from Meilunbio^®^ (Dalian, China). Recombinant Human Fibroblast Growth Factor 21 was purchased from Prime Gene^TM^ (Shanghai, China).

### RNA interference of ATG7 siRNA and FGF21 siRNA

Small interfering RNA (siRNA) sequences against ATG7, FGF21 and a scrambled control siRNA were designed and synthesized by SANTA CRUZ BIOTE CHNOLOGY, INC (ATG7 siRNA sc-41447; FGF21 siRNA sc-39484). siRNAs were transfected as previously described^53^. Protein expressions were analyzed by Western blot.

### Cell viability assay

Cell viability was detected by the 3-(4-5-dimethylthiazol-2-yl)-2,5-diphenyllte -trazolium bromide (MTT, Sigma–Aldrich) assay. Cells were treated with MTT (0.5 mg/ml) at 37 °C for 4 h. Then the insoluble formazan produced by viable cells was washed with PBS. 150 μl Dimethyl sulfoxide (DMSO) was added into each well for solubilizing the precipitated formazan, and the formazan solution was shock for 15 minutes and then incubated for 10 minutes in 37 °C. The absorbance was measured at 490 nm using a microplate reader (POLARstar Omega®, BMG, Germany).

### Measurement of glucose uptake

Glucose concentration in HK-2 cell medium supernatant was estimated using a commercial kit (Jiancheng Bioengineering Institute, Nanjing, China) by Glucose oxidase-peroxidase(GOD-POD) assay, and the absorbance was measured at 505 nm using a microplate reader (POLARstar Omega^®^, BMG, Germany). In addition, glucose uptake in HK-2 cells was tested by 2-(N-(7-Nitrobenz-2-oxa- 1,3-diazol- 4-yl) Amino)-2-Deoxyglucose (2-NBDG, KeyGEN BioTECH, nanjing, China), a fluorescent indicator. The fluorescence intensity immediately measured at an excitation/emission wavelength of 465/540 nm in a fluorescent microplate reader (POLARstar Omega®, BMG, Germany).

### Flow cytometry assay

The quantitative measurements of GLUT4 translocation from intracellular storage to the plasma membrane was assessed by flow cytometry. Essentially, HK-2 cells grown in 6-well plates and were treated as indicated. After washed by PBS, cells were digested with trypsin EDTA-free and terminated by F12/DMEM medium (10% serum, no phenol red). After centrifugation at 1000 rpm for 5 min, HK-2 cells were fixed with 4% paraformaldehyde in PBS for 20 min and then blocked with 5% bovine serum albumin (BSA) for 30 min at room temperature. After two washes, HK-2 cells were incubated with anti-rabbit GLUT4 (1:50, BOSTER, wuhan, China) for 30 min at room temperature, followed by incubation with FITC labeled goat anti-rabbit IgG (1:75, BOSTER, wuhan, China) for 30 min at room temperature. After extensively washing with PBS, HK-2 cells were resuspended in 500 µl PBS and analyzed on a flow cytometer (BD Accuri). The mean fluorescence intensity (MFI) was calculated using Flowjo Software Version 7.6.

### Western blot assay

Western blot analysis was performed as described previously^54^. Briefly, total proteins from the HK-2 cells and the rat kidney were extracted using RIPA lysis buffer (Biouniquer, BU-P0301). Protein concentration was quantified using a BCA Protein Assay Kit (keygen biotech co., LTD, Nanjing, China), and boiled for 5 minutes at 95 °C in a 5× loading buffer. Protein lysates (30 μg protein per sample) were loaded and separated on sodium dodecyl sulfate polyacrylamide gels (SDS-PAGE) and then electrophoretically transferred onto a polyvinylidene difluoride (PVDF) membrane at 200 mA for 1 hour. The membrane was blocked with 5% nonfat milk in Tris-buffered saline with Tween 20 (0.2%) for 1 h. After washing, the membranes were probed with primary antibodies overnight at 4 °C. The primary antibodies used are rabbit anti-LC3B (dilution 1:1000, proteintech^TM^, wuhan, China), rabbit anti-Beclin1 (dilution 1:1000, proteintech^TM^, wuhan, China), rabbit anti-p62 (1:1000, proteintech^TM^, wuhan, China), rabbit anti-pIRS-1ser307 (1:1000, proteintechTM, wuhan, China), rabbit anti-IRS-1 (1:1000, proteintech^TM^, wuhan, China), rabbit anti-p-AKT (1:1000, Affinity Biosciences, OH, USA), rabbit anti-AKT (1:1000, proteintech^TM^, wuhan, China), rabbit anti-FGF21 (1:1000, Abcam, USA), rabbit anti-GAPDH (1:1000, proteintech^TM^, wuhan, China). After 3 times of wash, the membranes were incubated with Goat anti-rabbit IgG (1:3500, Wuha Boster Biological Technology) for 1.5 h. The blot was detected by chemiluminescent detection systems with LumiGlo and Peroxide (1:1, BU), and the original gels were shown in supplementary file. Densitometric analysis of the images was performed with ImageJ software (NIH, Littleton, CO, USA).

### Monodansylcadaverine staining

HK-2 cells were seeded in 24-well cell culture plate (3 × 10^5^ cells per well). After treatment with PA for 24 hours, cells were stained with 50 μM Monodansylcadaverine (MDC, keygen biotech co., LTD, Nanjing, China)^55^ for 30 min at 37 °C. After washing with PBS, the cells were immediately visualized by a Laser scanning confocal microscope (Carl Zeiss, LSM700) at an excitation/emission (nm) of 355/512. The fluorescence intensity was analyzed and processed by ImageJ software.

### Animal and type 2 diabetic rat model

Adult male Sprague-Dawley rats, weighing 130–150 g, were obtained from Qinglongshan Lab Animal Ltd, Nanjing, China. Animal handling and experimental procedures were approved by the ethic committee of China Pharmaceutical University, in accordance with the Guidelines of Animal Experiment set by the Bureau of Sciences and Techniques of Jiangsu Province, China [NO.SYXK2007-0025].

Male Sprague-Dawley rats were fed a high fat diet (HFD) and a low dose of STZ to develop a rat model of type2 diabetes according to the method described by M.J. Reed *et al*.^56^ and Xiao-dong LIU *et al*.^7^. Rats were fed with a HFD (22 g/d) consisting of 10% saccharose, 10% lard, 10% sugar, 5% egg yolk powder, 0.5% cholesterol, 74.5% basal chow. The ordinary chow was constituted by 36% corn, 23% triturate wheat, 10% bran, 12% soy bean powder, 3% egg; 12% fish powder, 2% driedyeast, 1% of amixture of calcium bicarbonate, multi-vitamins and micro-elements. Both the normal chow and high-fat diet were purchased from Qinglongshan Lab Animal Ltd (Nanjing, China).

Rats were divided into three groups: control, untreated diabetes, and diabetes treated with PGE1. In the first 6 weeks, rats in each group were given a HFD alone with the normal group receiving regular chow only. On the beginning of 7th week, rats fed with a HFD received a single time intraperitoneal injection of Streptozotocin (STZ, 40 mg/kg, dissolved in pH 4.5 citrate buffer). Control rats received only an equivalent volume of citrate buffer. After the injection of STZ all groups maintained their original diets.

Rats on HFD with low STZ were recognized as diabetic while the fasting blood glucose (FBG) level reached16.7 mM after 1 week of STZ injection. In weeks 9–12, the interventions with PGE1 (20 μg/kg/d) were conducted, however, rats in the groups of normal and untreated diabetes were given an equal volume of saline solution containing 0.6% ethanol. A gain of body weight was monitored at an interval of 3 days.

### Biochemical assay

Fasting plasma insulin (FIN) were detected by rat insulin enzyme-linked immunosorbent assay kit (Halin Biological technology co., LTD, shanghai China). The index of evaluating insulin sensitivity was Homeostatic Model Assessment (HOMA-IR)^57,58^, which was calculated as FBG (mmol/L) × FIN (mU/L)/22.5. Free fatty acid (FFA), urine protein and creatinine in plasma as well as glucose and creatinine in urine were assayed following instructions of the kits provided by the Nanjing Jiancheng Bio engineering Institute (China). The creatinine clearance (Ccr) is calculated as follows Ccr = U × V/P (ml/min); V: The volume of urine per minute (ml/min) = Total urine volume(ml) ÷ (24 × 60)min; U: Urinary creatinine, umol/L; P: Serum/plasma creatinine, umol/L.

### Histological analysis and immunohistochemistry

Kidneys were fixed in 4% paraformaldehyde and embedded in paraffin. Paraffin sections were cut at 3 μm and deparaffinized for HE staining. Paraffin sections were dyed with Hematoxylin for 10 minutes, alcoholic eosin for 30 seconds respectively. Then the sections were treated with solution of xylene solution of carbolic acid for 30 seconds, encapsulated into slices and observed under inverted microscope.

Kidneys were fixed in 4% paraformaldehyde and embedded in paraffin. Paraffin sections were cut at 3μm and deparaffinized for immunoperoxidase staining. Sections were blocked with 3% hydrogen peroxide at room temperature for 10 minutes after antigen retrieval. Sections were blocked with skim milk for 10 minutes. The primary antibodies were used rabbit anti-pIRS-1ser307 (1:100, proteintechTM, wuhan, China), rabbit anti-LC3B (dilution 1:100, proteintechTM, wuhan, China), rabbit anti-FGF21 (1:500, Abcam, USA) were applied for 12 h at 4 °C. Secondary antibodies were incubated for 30 minutes. Then the horseradish enzyme labeled Streptomyces biotin solution was added for 20 minutes at 37 °C. DAB colored sections for 3–10 minutes, then washed. The sections were counterstained with hematoxylin before being examined under a light microscope. Optical density values were determined by Image Pro Plus 5.0.2 software.

### Statistics

Data are presented as means ± SE. Significant differences between and within multiple groups were examined using ANOVA for repeated measures, followed by Duncan’s multiple-range test. The Students *t* test was used to detect significant differences between two groups. *P* < 0.05 was considered statistically significant.

## Electronic supplementary material

Supplementary Information(original gel)
