# Supplementary material for: Inhibition of insulin resistance by PGE1 via autophagy-dependent FGF21 pathway in diabetic nephropathy
Source: Sci Rep. 2018 Jan 8;8:9. doi: 10.1038/s41598-017-18427-2 (PMC5758726; doi:10.1038/s41598-017-18427-2)
Supplement: Supplementary file 1 — Supplementary Information(original gel) [file 41598_2017_18427_MOESM1_ESM.pdf]

Inhibition of insulin resistance by PGE1 via autophagy-dependent FGF21 pathway in diabetic nephropathy

Wei Wei, Xing-rong An, Shi-Jie Jin, Xiao-Xue Li, Ming Xu

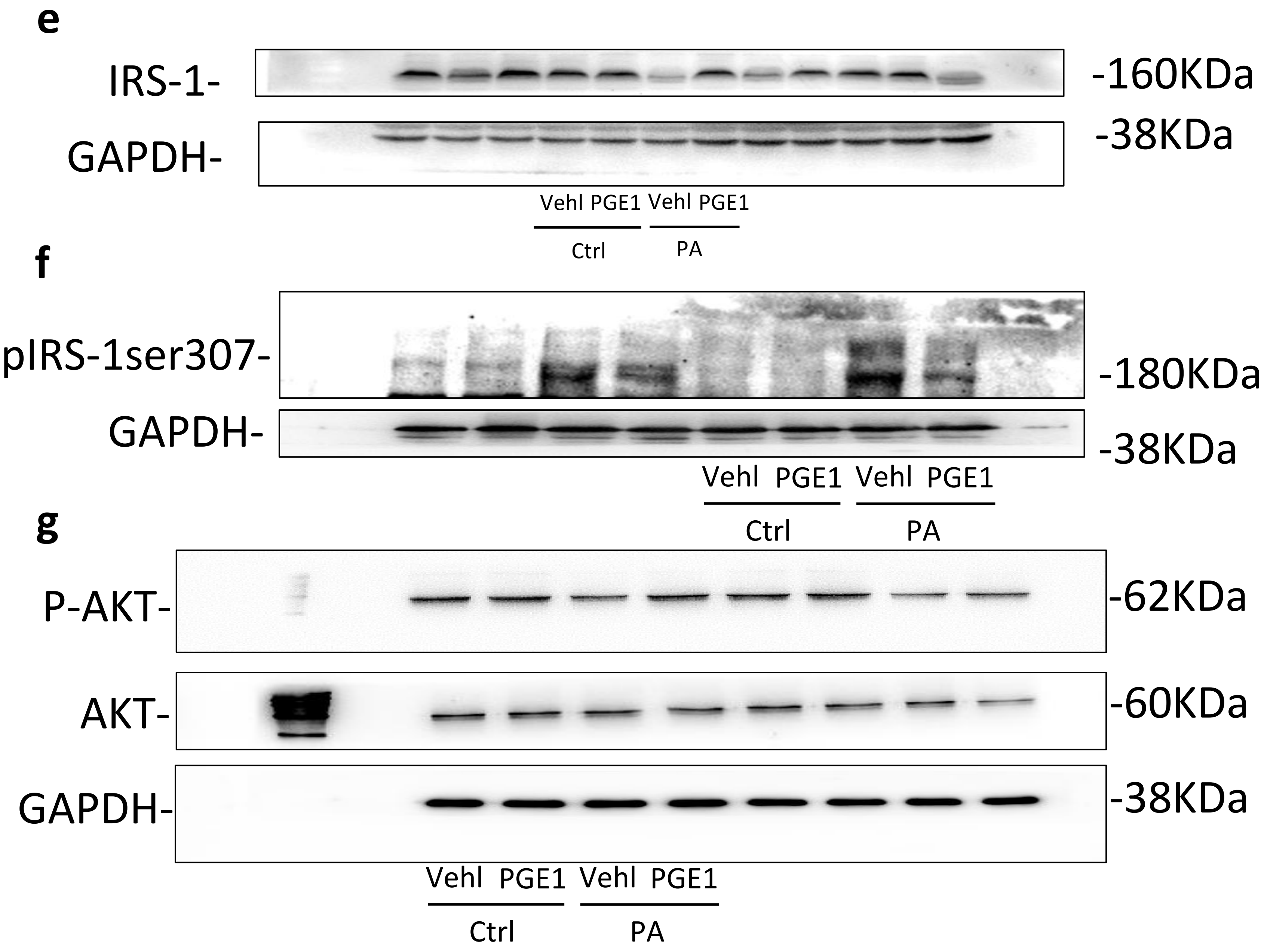

e
IRS-1-
-160KDa
GAPDH-
-38KDa
Vehl PGE1 Vehl PGE1
Ctrl
PA
f
pIRS-1ser307-
-180KDa
GAPDH-
-38KDa
Vehl PGE1 Vehl PGE1
Ctrl
PA
g
P-AKT-
-62KDa
AKT-
-60KDa
GAPDH-
-38KDa
Vehl PGE1 Vehl PGE1
Ctrl
PA

Figure 1

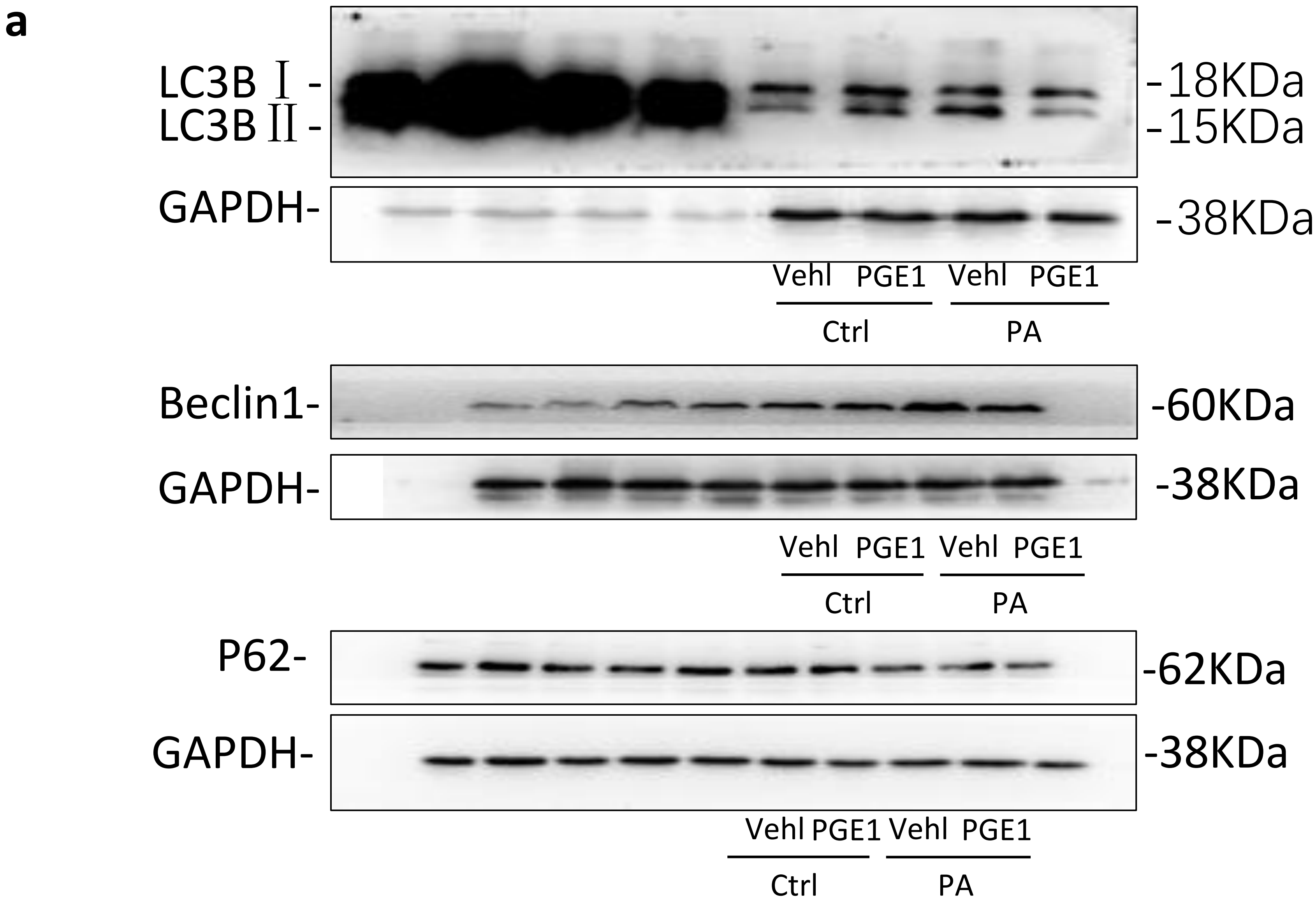
a
LC3B Ⅰ -
LC3B Ⅱ -
-18KDa
-15KDa
GAPDH-
-38KDa
Vehl
PGE1
Vehl
PGE1
Ctrl
PA
Beclin1-
-60KDa
GAPDH-
-38KDa
Vehl
PGE1
Vehl
PGE1
Ctrl
PA
P62-
-62KDa
GAPDH-
-38KDa
Vehl
PGE1
Vehl
PGE1
Ctrl
PA

Figure 2

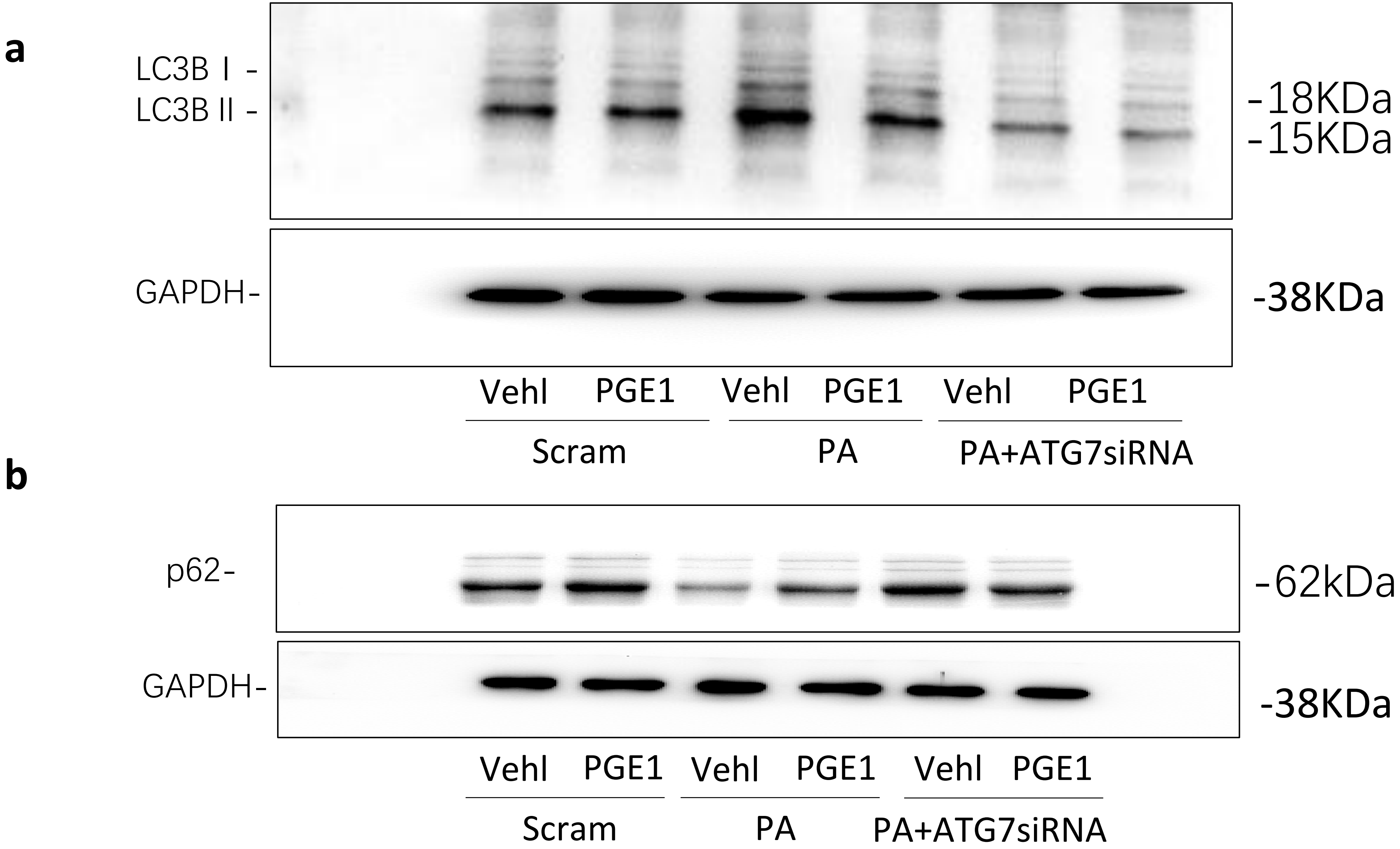
a
LC3B I -
LC3B II -
-18KDa
-15KDa
GAPDH-
-38KDa
Vehl
PGE1
Vehl
PGE1
Vehl
PGE1
Scram
PA
PA+ATG7siRNA
b
p62-
-62kDa
GAPDH-
-38KDa
Vehl
PGE1
Vehl
PGE1
Vehl
PGE1
Scram
PA
PA+ATG7siRNA

Figure 3

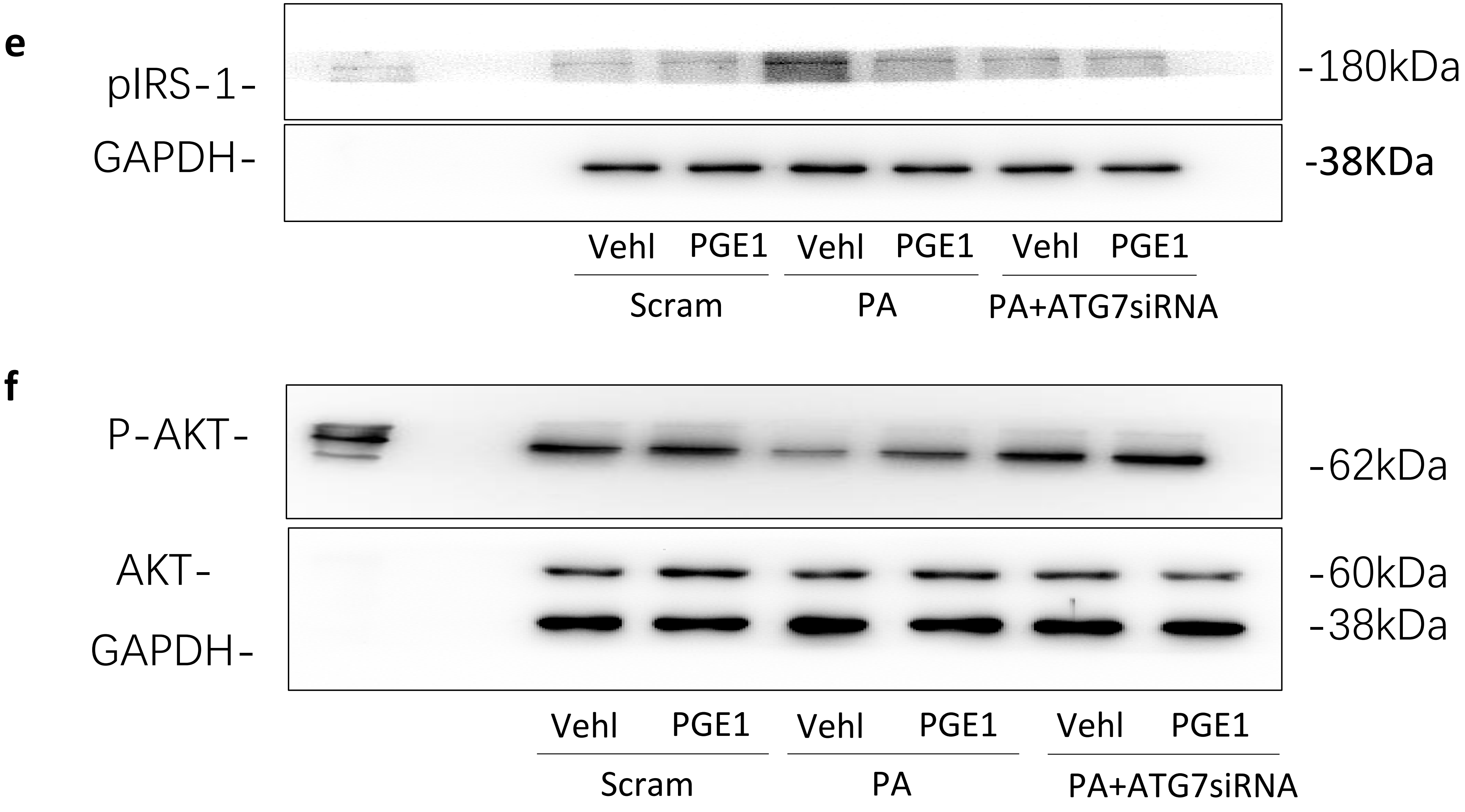
e
pIRS-1-
-180kDa
GAPDH-
-38KDa
Vehl PGE1 Vehl PGE1 Vehl PGE1
Scram
PA
PA+ATG7siRNA
f
P-AKT-
-62kDa
AKT-
-60kDa
-38kDa
GAPDH-
Vehl PGE1 Vehl PGE1 Vehl PGE1
Scram
PA
PA+ATG7siRNA

Figure 3

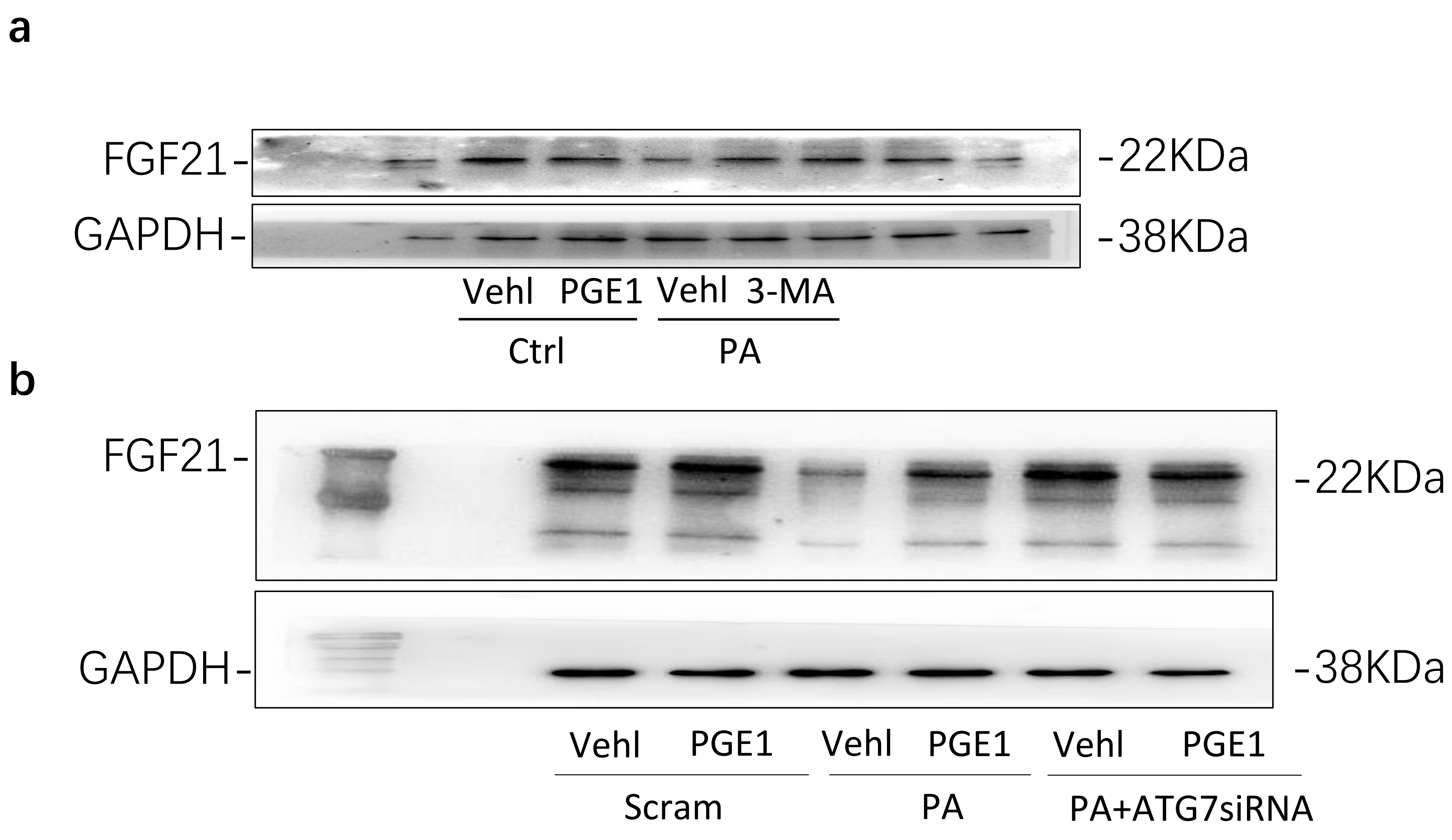
a
FGF21-
GAPDH-
-22KDa
-38KDa
Vehl PGE1 Vehl 3-MA
Ctrl
PA
b
FGF21-
GAPDH-
-22KDa
-38KDa
Vehl PGE1 Vehl PGE1 Vehl PGE1
Scram
PA
PA+ATG7siRNA

Figure 4

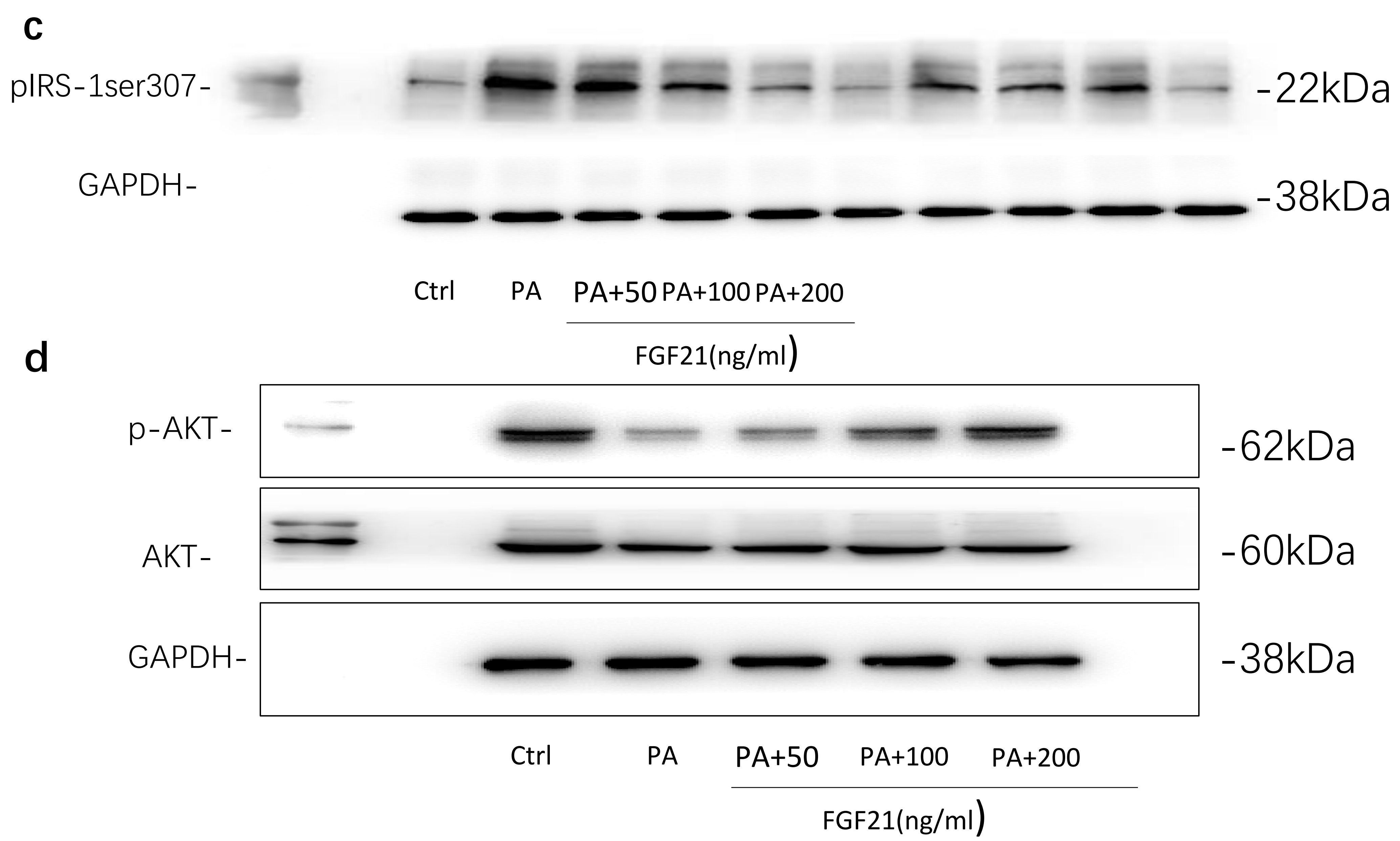
c
pIRS-1ser307-
-22kDa
GAPDH-
-38kDa
Ctrl
PA
PA+50
PA+100
PA+200
FGF21(ng/ml)
d
p-AKT-
-62kDa
AKT-
-60kDa
GAPDH-
-38kDa
Ctrl
PA
PA+50
PA+100
PA+200
FGF21(ng/ml)

Figure 5

c

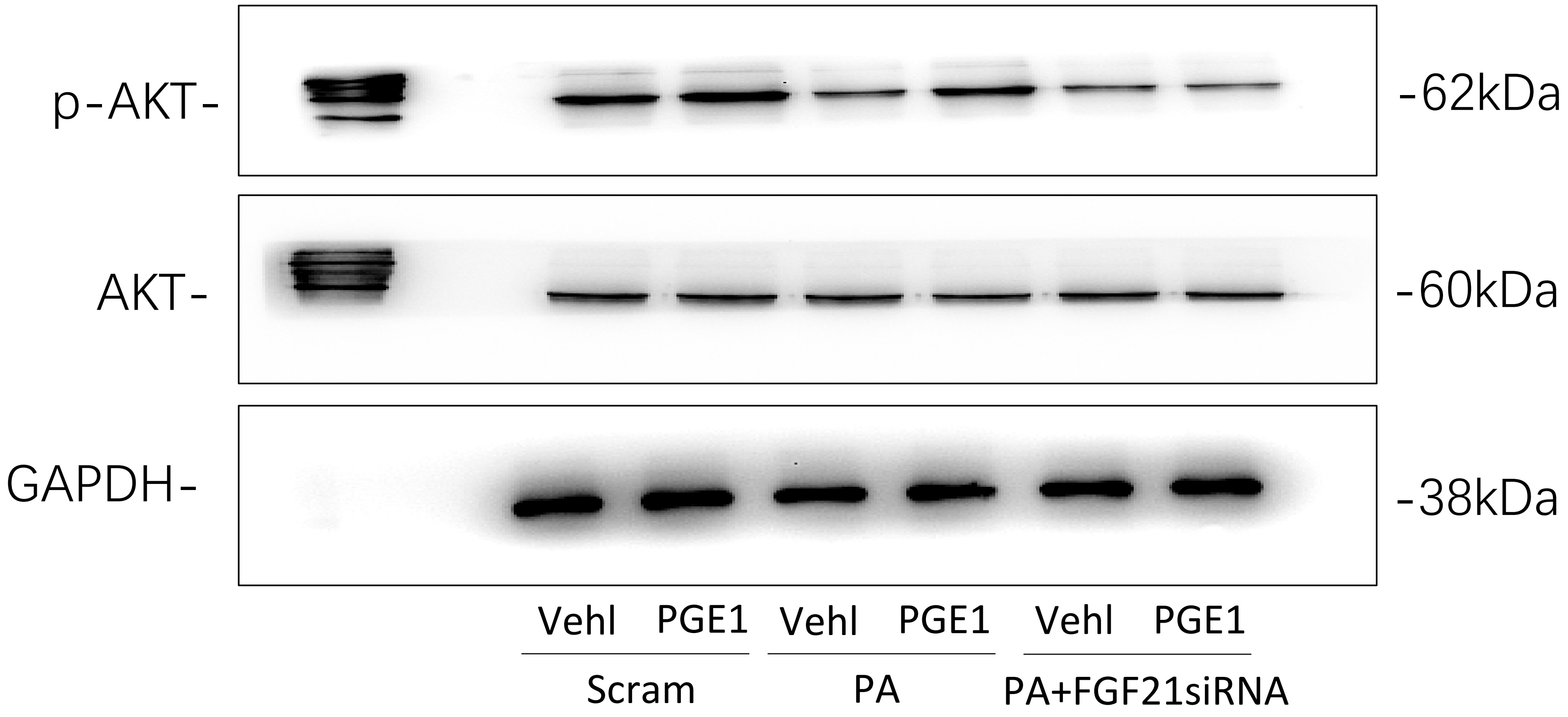
p-AKT-
AKT-
GAPDH-
-62kDa
-60kDa
-38kDa
Vehl
PGE1
Vehl
PGE1
Vehl
PGE1
Scram
PA
PA+FGF21siRNA

Figure 6

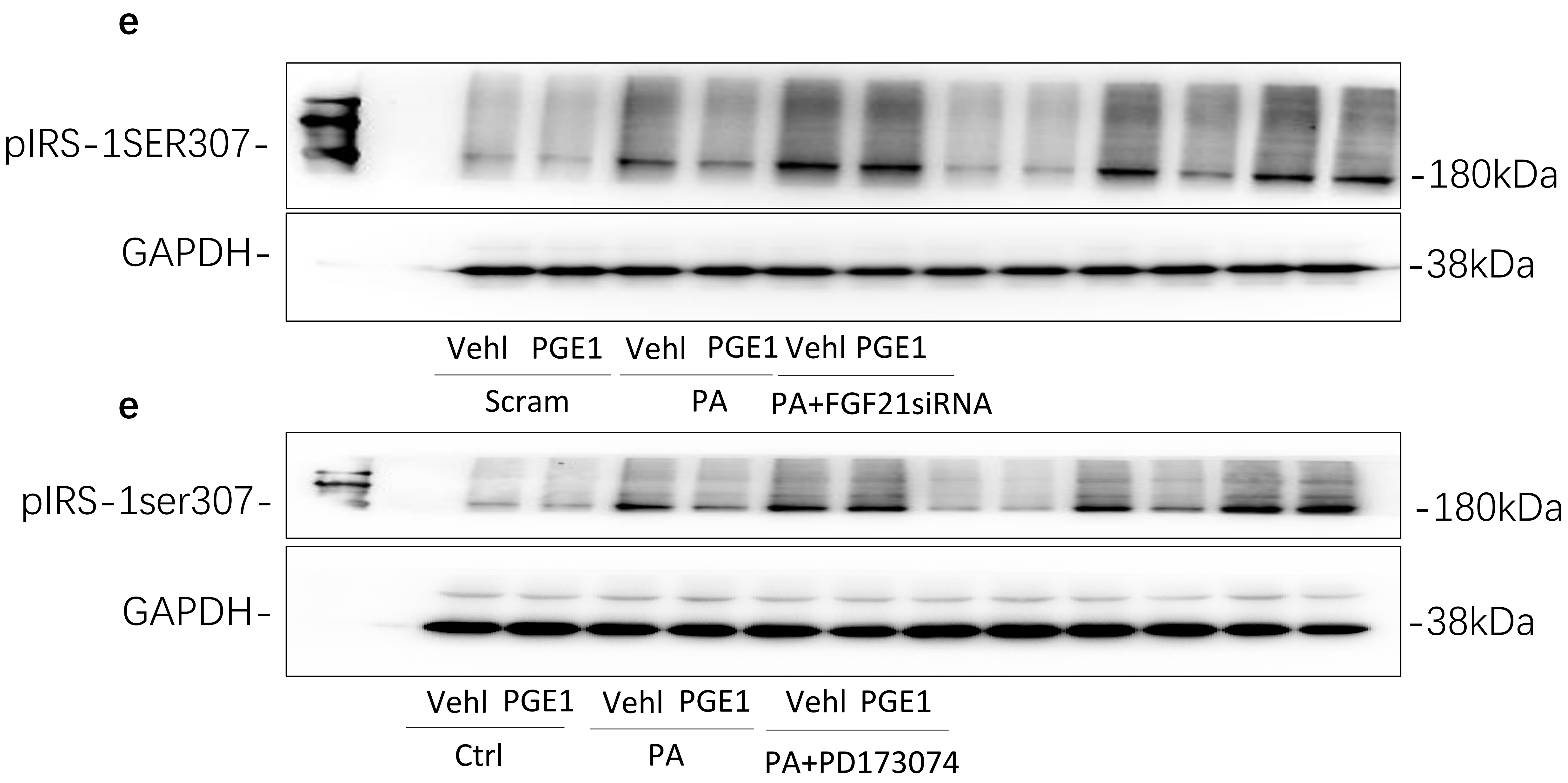
e
pIRS-1SER307-
-180kDa
GAPDH-
-38kDa
Vehl PGE1 Vehl PGE1 Vehl PGE1
Scram
PA
PA+FGF21siRNA
e
pIRS-1ser307-
-180kDa
GAPDH-
-38kDa
Vehl PGE1 Vehl PGE1 Vehl PGE1
Ctrl
PA
PA+PD173074

Figure 6

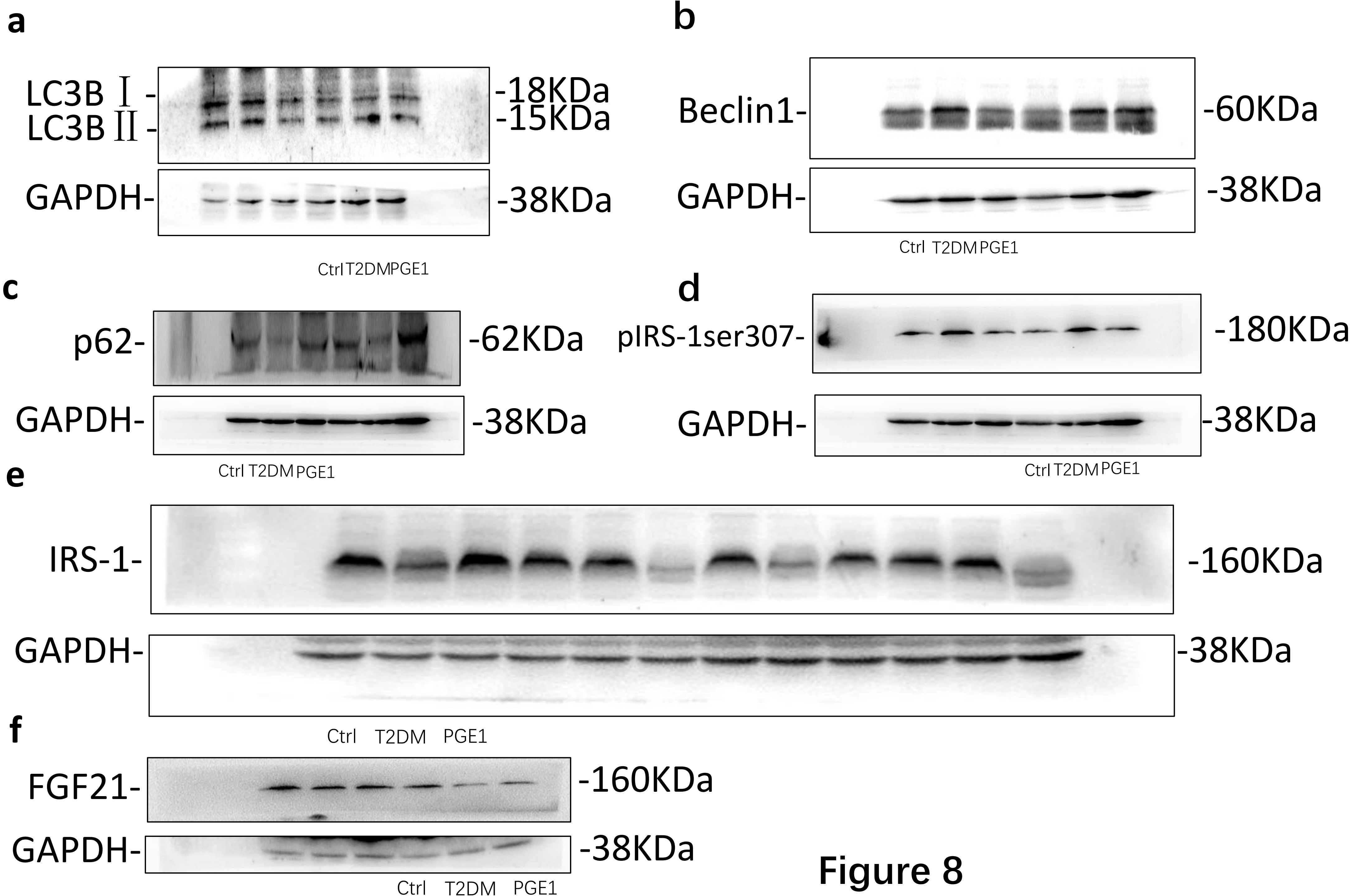
a
LC3B Ⅰ -
LC3B Ⅱ -
-18KDa
-15KDa
GAPDH-
-38KDa
Ctrl T2DM PGE1
b
Beclin1-
-60KDa
GAPDH-
-38KDa
Ctrl T2DM PGE1
c
p62-
-62KDa
GAPDH-
-38KDa
Ctrl T2DM PGE1
d
pIRS-1ser307-
-180KDa
GAPDH-
-38KDa
Ctrl T2DM PGE1
e
IRS-1-
-160KDa
GAPDH-
-38KDa
Ctrl T2DM PGE1
f
FGF21-
-160KDa
GAPDH-
-38KDa
Ctrl T2DM PGE1

Figure 8
